# Research on expansion and classification of imbalanced data based on SMOTE algorithm

**DOI:** 10.1038/s41598-021-03430-5

**Published:** 2021-12-15

**Authors:** Shujuan Wang, Yuntao Dai, Jihong Shen, Jingxue Xuan

**Affiliations:** 1 College of Mathematical Sciences, Harbin Engineering University, Harbin, 150001 China; 2 College of Science, Qiqihar University, Qiqihar, 161006 China

**Keywords:** Applied mathematics, Scientific data

## Abstract

With the development of artificial intelligence, big data classification technology provides the advantageous help for the medicine auxiliary diagnosis research. While due to the different conditions in the different sample collection, the medical big data is often imbalanced. The class-imbalance problem has been reported as a serious obstacle to the classification performance of many standard learning algorithms. SMOTE algorithm could be used to generate sample points randomly to improve imbalance rate, but its application is affected by the marginalization generation and blindness of parameter selection. Focusing on this problem, an improved SMOTE algorithm based on Normal distribution is proposed in this paper, so that the new sample points are distributed closer to the center of the minority sample with a higher probability to avoid the marginalization of the expanded data. Experiments show that the classification effect is better when use proposed algorithm to expand the imbalanced dataset of Pima, WDBC, WPBC, Ionosphere and Breast-cancer-wisconsin than the original SMOTE algorithm. In addition, the parameter selection of the proposed algorithm is analyzed and it is found that the classification effect is the best when the distribution characteristics of the original data was maintained best by selecting appropriate parameters in our designed experiments.

## Introduction

Imbalanced data typically refers to a problem with classification problems where the classes are not represented equally, including binary classification problems as well as multi-class classification problems^1^. For multi-classification problem, the category with more data samples is called majority category, while the category with less data samples is called minority category. For binary classification problems, categories with more data samples are called negative samples, and categories with less data samples are called positive samples^2^. In recent years, the classification of imbalanced data sets has been widely concerned. Class distributions that are highly skewed tend to bias the results of a machine learning or data mining algorithm, because the performance index used by machine learners^3–8^ is usually the overall accuracy^9^. For example, there are 90 normal samples in disease classification and only 10 diseased samples. Even if all diseased samples are misclassified, the accuracy of the model is still 90%, but the sensitivity and specificity are both 0. So, in a practical sense, the characteristics of the data can’t be accurately learned by the model, and the samples can’t be accurately classification. For the patients, misdiagnosis has a great impact and will have serious consequences.

Nowadays, the classification of imbalanced data sets has become a hot issue in data mining^10^, and has been thoroughly studied by scholars from the data layer and the algorithm layer.

From the algorithm layer, the classification performance of the algorithm is improved by the algorithm structure design. Galar proposed a new ensemble algorithm (EUSBoost) based on RUSBoost, which combines random under-sampling with enhancement algorithm, effectively avoiding over fitting^11^. In Datta’s paper, a Near-Bayesian Support Vector Machine (NBSVM) is developed focused on the philosophies of decision boundary shift and unequal regularization costs^12^. Qian proposed a resampling integration algorithm based on the classification problems for imbalanced datasets. In the method, the majority classes are under-sampled and minority classes are oversampled^13^. Chen proposed a Long Short-Term Memory-based Property and Quantity Dependent Optimization (LSTM.PQDO) method. The method realizes the dynamic optimization of the resampling proportion and overcome the difficulties of imbalanced datasets^14^. Hou proposed a time-varying optimization module to optimize the results of special periods and effectively eliminate imbalances^15^.

The main idea based on the data level is to construct the minority samples to increase the imbalance rate^12^ (The ratio of the number of minority samples to the number of majority classes). Chawla proposed the SMOTE (Synthetic Minority Over-sampling Technique) algorithm^16^. Blagus investigated the properties of SMOTE from a theoretical and empirical point of view, using simulated and real high-dimensional data^17^. In order to solve the noise problem generated by the SMOTE algorithm, Mi introduced the classification performance of support vector machines and proposed an imbalanced data classification method based on active learning SMOTE^18^. Seo uses machine learning algorithms to find effective SMOTE ratios for rare categories (such as U2R, R2L, and Probe)^19^. A novel ensemble method, called Bagging of Extrapolation Borderline-SMOTE SVM (BEBS), has been proposed in dealing with Imbalanced Data Learning (IDL) problems^20^. Based on the ensemble algorithm, Yang proposed anovel intelligent classification model based on SMOTE and ensemble learning to classify railway signal equipment faults^21^. Douzas presented G-SMOTE, a new over-sampling algorithm, that extends the SMOTE data generation mechanism. G-SMOTE selects a safe radius around each minority of clustering algorithm^22^. Ma proposed the CURE-SMOTE (Combination of Clustering Using Representatives Synthetic Minority Over-sampling Technique) algorithm^23^. Experiments on the UCI imbalanced data show that the original Synthetic Minority Over-sampling Technique is effectively enhanced by the use of the combination of clustering using representative algorithm. In Prusty’s paper, SMOTE has been modified to Weighted-SMOTE (WSMOTE) where over-sampling of each minority data sample is carried out based on the weight assigned to it^24^. Xwl proposed the LR-SMOTE algorithm. The algorithm makes the newly generated samples close to the center of the sample, avoiding generating outlier samples or changing the distribution of data sets^25^. Fernandez reflect on the SMOTE journey, discuss the current state of affairs with SMOTE, its applications, and also identify the next set of challenges to extend SMOTE for big data problems^26^. Majzoub proposed Hbrid Custering Affinitive Borderline SMOTE (HCAB-SMOTE). It manages to minimize the number of generated instances while improving the classification accuracy^27^. Chen introduced relative density to measure the local density of each minority sample, and divides non-noise minority samples into boundary samples and safety samples adaptively according to the distinguishing characteristics of relative density, which effectively enhances the separability of the boundary^28^.

SMOTE algorithm can improve the classification effect of imbalanced data by randomly generating new minority sample points to increase the imbalance rate to a certain extent. However, the SMOTE algorithm has two shortcomings. On one hand, the SMOTE algorithm generates the minority sample points by random linear interpolation between the minority sample points and their neighbors, so the edge points of minority samples may produce distribution marginalization. On the other hand, the value of k (the number of nearest points selected when generate new points according to a certain minority sample point) needs to be set manually when the SMOTE algorithm performs data expansion. Based on the SMOTE algorithm and the idea of Normal distribution, this paper proposes a novel data expansion algorithm for imbalanced data sets. The Uniform random distribution in the original SMOTE algorithm was replaced by the Normal random distribution, so that the newly generated sample points are distributed near the center of the minority sample with a higher probability, which can avoid the marginalization of the expanded data. Then, this paper analyzes the parameter selection of the proposed algorithm. Appropriate parameter selection can make the expanded data maintain the distribution characteristics (inter-class distance and sample variance) of the original data. The experimental results show that the classification effect of the random forest after data expanded by proposed algorithm is better than the original SMOTE on the imbalanced data sets of Pima, WDBC, WPBC, Ionosphere and Breast-cancer-wisconsin.

## Methods

### SMOTE algorithm

SMOTE (Synthetic Minority Over-sampling Technique) algorithm is an extended algorithm for imbalanced data proposed by Chawla^16^. In essence, SMOTE algorithm obtains new samples by random linear interpolation between a few samples and their neighboring samples. The data imbalance ratio is increased by generating a certain number of artificial minority samples, so that the classification effect of the imbalanced data set is improved^18^. The specific process of SMOTE is as follows.

Step 1. For each minority sample $x_{i} \left( {i = 1,2, \ldots ,n} \right)$, calculate its distance to other samples in minority sample according to certain rules to obtain its $k$ nearest neighbors.

Step 2. According to the over-sampling magnification, the random $m$ nearest neighbors, as a subset of $k$ nearest neighbors set, of each sample $x_{i}$ are selected and denoted as $x_{ij} \left( {j = 1,2, \ldots ,m} \right)$, then an artificially constructed minority sample $p_{ij}$ is calculated by Eq. (1).

1 $$p_{ij} = x_{i} + rand(0,1) \times (x_{ij} - x_{i} )$$

where $rand(0,1)$ is a random number uniformly distributed within the range of [0,1]. The operation of formula (1) is stopped until the fused data reaches a certain imbalance ratio.

### Motivation

Marginalization may occur when the SMOTE algorithm constructs data. If a positive (minority) sample point near to the distribution edge of the positive sample set, the "artificial" sample points generated by the positive sample point and adjacent sample points may also be on this edge and become more and more marginalized^23^. As a result, the boundaries between positive and negative (majority sample) samples are blurred. Therefore, an improved SMOTE algorithm based on the Normal distribution^29,30^ is proposed in this paper, and the distribution of the generated data samples is controlled by appropriate parameters selection.

The $rand\left( {0,1} \right)$ denotes a random number falling in the interval of (0,1) with equally probability, so the generated sample points will be evenly distributed between the sample point $x_{i}$ and its neighbor $x_{ij} \left( {j = 1,2, \ldots ,m} \right)$ in Eq. (1), which will lead to the phenomenon of marginalization of the expanded data when the sample point $x_{i}$ is near to or on the edge of the minority sample. While, if the Uniform distribution random number $rand\left( {0,1} \right)$ is replaced by a Normal distribution random number $randn$, and the minority sample center is used to substitute $x_{ij}$, then the expanded points will be distributed near the sample center with a higher probability (details in Eq. 5). Where $randn$ denotes a random number obeying Normal distribution with the mean value of $\mu = 1$ and standard deviation of $\sigma$ (adjustable). And the number $p = randn$ has the following distribution characteristics.

$$\begin{aligned} & P\left( {\mu - \sigma \le p \le \mu + \sigma } \right) \approx 0.6826 \\ & P\left( {\mu - 2\sigma \le p \le \mu + 2\sigma } \right) \approx 0.9544,\,\,P\left( {\mu - 3\sigma \le p \le \mu + 3\sigma } \right) \approx 0.9974. \\ \end{aligned}$$

The core of the improved SMOTE algorithm based on the Normal distribution is to make the generated new samples gather towards to the center of minority samples with a high probability, and could preserve the statistic characteristics of the original minority by proper parameter selection.

### Improved algorithm design

The process of improved SMOTE algorithm based on Normal distribution is as follows.

Step 1. Standardize the original data by Eq. (2) to avoid errors caused by different dimensions.

2 $$x_{ij}^{\prime } = \frac{{x_{ij} - x_{j\min } }}{{x_{j\max } - x_{j\min } }}$$

where $x_{ij}$ is the $i$-th sample point under the $j$-th feature of the original data, $x_{j\min }$ and $x_{j\max }$ are the minimum and maximum value in the $j$-th feature respectively.

Step 2. Calculate the center point $x_{center}^{\prime }$ of minority samples.

3 $$x_{center}^{\prime } = \left( {\frac{1}{n}\sum\limits_{i = 1}^{n} {x_{i1}^{\prime } } ,\frac{1}{n}\sum\limits_{i = 1}^{n} {x_{i2}^{\prime } } , \ldots ,\frac{1}{n}\sum\limits_{i = 1}^{n} {x_{ir}^{\prime } } } \right)$$

where $n$ is the total number of samples in minority samples, and $r$ is the number of features in minority samples.

Step 3. Estimate Normal distribution of $n \times 1$ dimensional normalized minority samples under each feature. Let $\sigma_{{0}}$ denote the standard deviation vector of the data set of the minority normalized samples.

4 $$\sigma_{0} = \left( {\sigma_{1}^{0} ,\sigma_{2}^{0} , \ldots ,\sigma_{r}^{0} } \right)$$

where $\sigma_{i}^{0} (i = 1,2, \ldots ,r)$ is the standard deviation of the $i$-th feature.

Step 4. Synthesis of new samples based on interpolation formula (5).

5 $$p_{i} = x_{i}^{\prime } + f\left( x \right) \cdot (x_{center}^{\prime } - x_{i}^{\prime } )$$

where $p_{i} \left( {i = 1,2, \ldots n} \right)$ is a newly generated minority sample. According to Eq. (5), it can be known that the main control part for data generation is $f\left( x \right)$. When the value of $f\left( x \right)$ is 1, $p_{i}$ is the minority sample center $x_{center}^{\prime }$. If $f\left( x \right)$ takes the values near to 1 with a higher probability, then the expanded minority samples will be closer to the center point $x_{center}^{\prime }$. Let $f\left( x \right)$ is a random number obeying Normal distribution with mean value of $\mu { = 1}$ and standard deviation $\sigma$. Then if take $\sigma { = }{{\sigma_{{0}} } \mathord{\left/ {\vphantom {{\sigma_{{0}} } 3}} \right. \kern-\nulldelimiterspace} 3}$, the value of $f\left( x \right)$ will appear in the interval of $\left( {1 - \sigma_{{0}} ,1 + \sigma_{{0}} } \right)$ with a probability of $99.74\%$ and the interval of $\left( {1 - {{\sigma_{{0}} } \mathord{\left/ {\vphantom {{\sigma_{{0}} } 3}} \right. \kern-\nulldelimiterspace} 3},1 + {{\sigma_{{0}} } \mathord{\left/ {\vphantom {{\sigma_{{0}} } 3}} \right. \kern-\nulldelimiterspace} 3}} \right)$ with the probability of $68.26\%$.

Step 5. The expansion stops until the imbalance ratio reaches 0.7. Ma conducted an extended experiment on 5 imbalanced data sets including Breast-cancer-wisconsin in the UCI database^23^. The experimental results showed that when the imbalanced ratio reached 0.7, the corresponding classification effect was better. So, we choose 0.7 as a threshold value of imbalance ratio to judge whether the expansion is enough.

Step 6. The newly generated minority data is fused with the original minority data.

**Figure 1:**
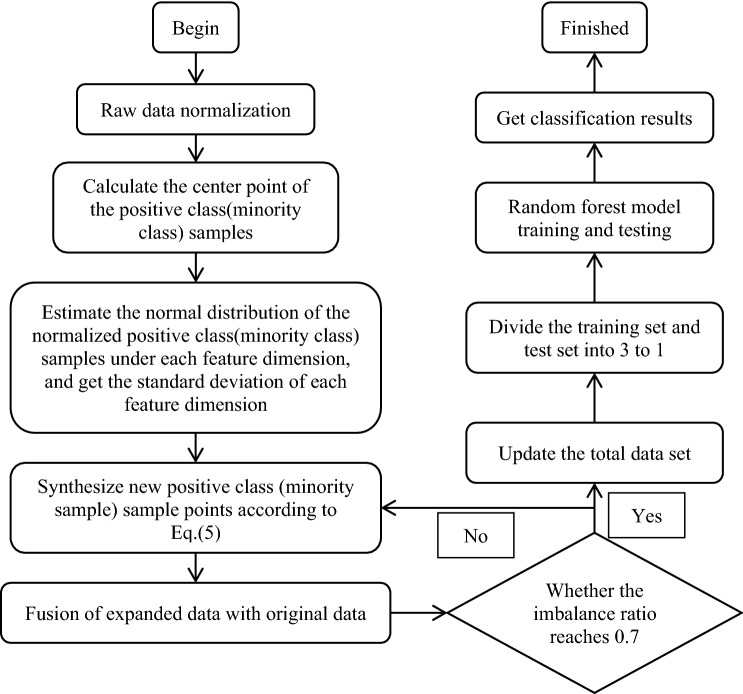
Improved algorithm flow chart.

The flow chart of the improved SMOTE algorithm based on Normal distribution is shown in Fig. 1.

## Classification method and evaluation index

### Random Forest algorithm

With the rapid development of the field of machine learning, random forest is widely used because of their high error tolerant performance and strong classification performance^16^. Traditional random forest algorithms are used to handle balanced data sets, but imbalanced data sets are more common, especially in practical problems. Random Forest (RF)^31–34^ is a bagging ensemble learning algorithm proposed by Leo Breiman in 2001. Multiple decision trees are constructed and combined to complete a learning task in parallel, and the final prediction and classification results are obtained by voting^35^. The process of the random forest is as follows.

- Step 1. The data is randomly divided into two sets, training set and test set.
- Step 2. During training, these data are randomly stratified by sampling them into *K* parts with *K*-fold cross-validation.
- Step 3. The bootstrap method is used to randomly extract a training set from the training set in *K*-fold cross-validation for each decision tree.
- Step 4. $h$ features are randomly selected from the $r$ features of each subnode in the decision tree as split attribute sets.
- Step 5. $N$ decision trees trained in parallel constructed as random forest models.
- Step 6. Based on the principle that the majority wins the minority, random forest vote to obtainthe last experiment results.
- Step 7. fivefold cross-validation is used in experiments, and the average of the accuracy of the validation set is calculated.

### Experimental evaluation index

The evaluation indexes *AUC*, *F*-value, *G*-value and *OOB*_error will be involved in this paper introduced in Eqs. (6)–(10). Suppose the data is divided into two categories, positive and negative, the confusion matrix is introduced in Table 1.

**Table 1:** Two-class confusion matrix.

	Predicted to be positive	Predicted as negative
Actually positive	*TP*	*FN*
Actually negative	*FP*	*TN*

Classification *Accuracy* (*AUC*):

6 $$AUC = \frac{TP + TN}{{TP + TN + FP + FN}}$$

*AUC* represents the ratio of the sum of samples that are correctly classified to the total number of samples. Generally, the higher the *AUC* value is, the better the classification effect of the model is.

*F*-value:

7 $$F = \frac{{(1 + \beta^{2} ) \times Sentivity \times Precision}}{{\beta^{2} \times {Sentivity + Precision}}}$$

where $\beta \in \left( {0,1} \right]$, but $\beta$ is generally taken to be 1. And

8 $$Sentivity = \frac{TP}{{TP + FN}},\;Precision = \frac{TP}{{TP + FP}}$$

*F*-value is an index for evaluating the classification performance of imbalanced sets from the perspective of positive samples. The higher the *F*-value is, the better the classification effect of the model is.

*G* (*G*eometric Mean of the True Rates) values:

9 $$G = \sqrt {Sensitivity \times Specificity}$$

where

$$Specificity = \frac{TN}{{FP + TN}}$$

A high *G*-value indicates that random forests are better at classifying imbalanced data.

*OOB*_error (Out of Bag error) values:

10 $$OOB\_error = \frac{{\sum\limits_{i = 1}^{ntree} {OOB\_error_{i} } }}{ntree}$$

where $ntree$ is the number of decision trees, and *OOB*_error of the overall sample data is the arithmetic mean of the out of bag error for each decision tree. The smaller the *OOB*_error, the better the classification effect of the model.

## Simulation experiment

### Experimental environment

The experimental data are derived from five data sets in the UCI (University of California Irvine) database, that is Pima, WPBC, Breast-cancer-wisconsin, WDBC, and Ionosphere dataset. The specific information of these datasets is summarized in Table 2. The hardware configuration is Intel(R) Core(TM) i5-3210 M, and the processing speeds of CPU and RAM are 2.50 GHz and 4.00 GB respectively. The random forest model is implemented using the Python 3.7 language package, and the improved SMOTE algorithm based on the Normal distribution is jointly implemented by Python, Excel, and SPSS. SPSS is used to estimate the normal distribution of each column of characteristic data and obtain the variance of the Normal distribution. The version of SPSS used in this paper is IBM SPSS Statistics 23.lnk. For the random forest model training, fivefold stratified cross validation is used to prevent overfitting. The number of features trained in each decision tree is generated based on the empirical formula $h = \log_{2}^{r} + 1$. When each decision tree is split, the Gini index is used to select the best features. To simulate the actual situation appropriately and preserve the degree of imbalance of the original data, the training set and testing set were divided using stratified random sampling at a ratio of 3:1.

**Table 2:** Characteristic description of raw imbalance data.

Data set	Sample size	Positive class	Negative class	Characteristic number	Imbalance ratio
Pima	768	268	500	8	0.536:1
WPBC	194	45	149	33	0.3020:1
WDBC	569	212	357	30	0.5938:1
Ionosphere	351	126	225	34	0.56:1
Breast-cancer-wisconsin	683	239	444	9	0.5383:1

### Numerical experiment

**Part 1**. The comparative experiment between the proposed algorithm and original SMOTE algorithm. The two algorithms are used to expand the 5 imbalance data sets respectively, and the expanding stops when the imbalance ratios reach 0.7. Random forests are then used to classify the extended data and original data to make the comparison. In order to obtain more scientific and reasonable experimental results, each data set is extended 5 times by both the SMOTE algorithm and the proposed algorithm. The average value of these 5 classification results is taken as the final experimental result (the same for experiments in Part 2).

**Part 2**. The influence of different parameters on classification result. When the proposed algorithm is used for expansion, the three values of standard deviation of the Normal distribution in Eq. (5) are considered, that is $\sigma = \sigma_{0}$, $\sigma = {{2\sigma_{0} } \mathord{\left/ {\vphantom {{2\sigma_{0} } 3}} \right. \kern-\nulldelimiterspace} 3}$ and $\sigma = {{\sigma_{0} } \mathord{\left/ {\vphantom {{\sigma_{0} } 3}} \right. \kern-\nulldelimiterspace} 3}$, where $\sigma_{0}$ is the standard deviation of the original minority normalized data. Random forest is used to classify the expanded data based on different parameters.

**Part 3**. The analysis of parameter selection according to inter-class distance and sample variance. Based on the classification results in part2, the inter-class distance and sample variance of the original data set and the fused data set are calculated. The inter-class distance is obtained by calculating the Euclidean distance between the center points of the majority sample and the minority sample.

## Results

### Part 1 experimental results

**Figure 2:**
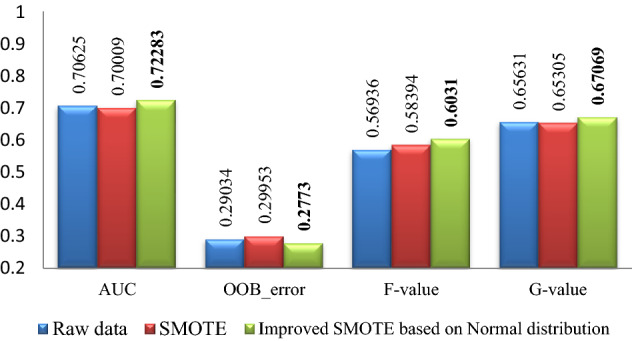
Pima experimental result graph.

**Figure 3:**
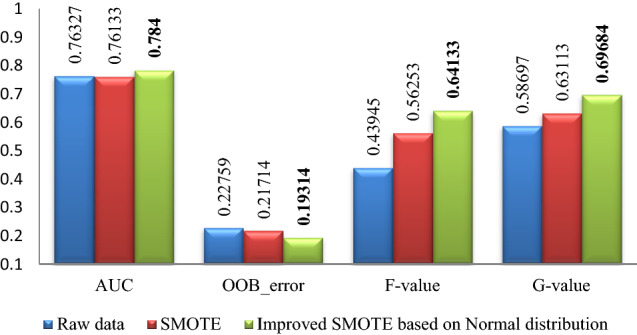
WPBC experimental result graph.

**Figure 4:**
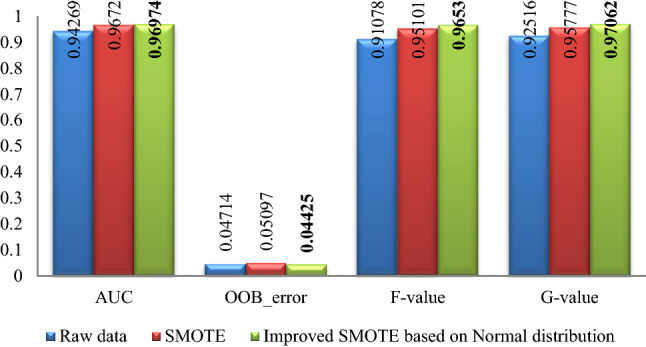
Breast-cancer-wisconsin experimental result graph.

**Figure 5:**
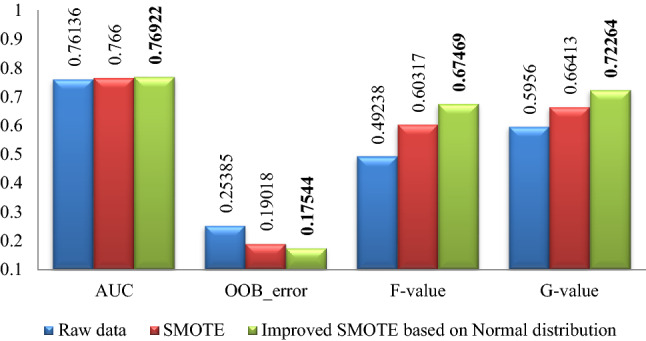
Ionosphere experimental result graph.

**Figure 6:**
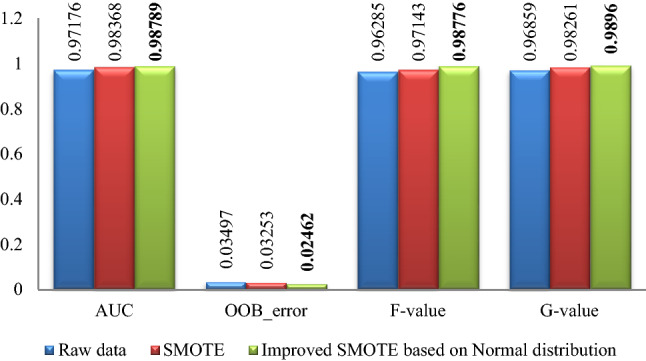
WDBC experimental result graph.

The experimental results of Pima, WDBC, WPBC, Ionosphere and Breast-cancer-wisconsin data sets are shown in Figs. 2, 3, 4, 5, 6. Where bold font indicates the better experimental results.

From Figs. 2, 3, 4, 5, 6, it is clear that the classification effect of each imbalance dataset extended by improved SMOTE is better than other conditions according to *AUC*, *OOB*_error, *F*-value and *G*-value, especially, Ionosphere and WPBC dataset are involved. Compared with the condition of original unexpanded data and the data expanded by the SMOTE algorithm, the classification effect of WPBC dataset after expanded by improved SMOTE algorithm shows an increase in classification accuracy by 2.073% and 2.267%, respectively; *OOB*_error value decreased by 3.445% and 2.4%; *F*-value increased by 20.188% and 7.88%; *G*-value increased by 10.987% and 6.571%. For the Ionosphere dataset, the classification effect after expanded by the improved SMOTE shows a 7.152% increase on *F*-value and 5.851% increase on the *G*-value than that condition based on original SMOTE.

In addition, the CURE-SMOTE algorithm is used to expand the Breast-cancer-wisconsin dataset and random forest is used to do classification in ^23^. The classification experimental results are 0.9621 (*AUC*), 0.9511 (*F*-value), 0.9621 (*G*-value) and 0.0427 (*OOB*_error) respectively. While the classification experimental results of the improved algorithm in this paper on the Breast-cancer-wisconsin dataset are 0.9697 (*AUC*), 0.9653 (*F*-value), 0.9706 (*G*-value) and 0.0443 (*OOB*_error). On the whole, it is not difficult to find that the improved algorithm shows a better classification effect on the index value.

### Part 2 experimental results

**Figure 7:**
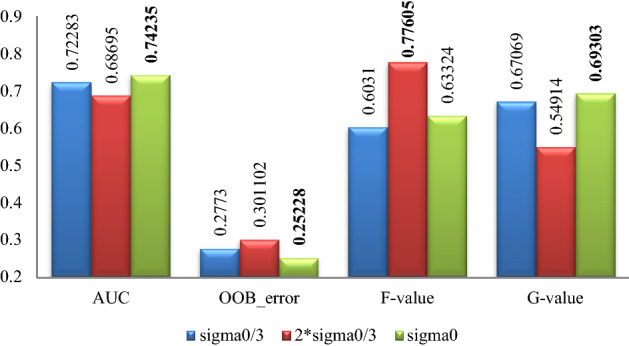
Pima experimental result graph.

**Figure 8:**
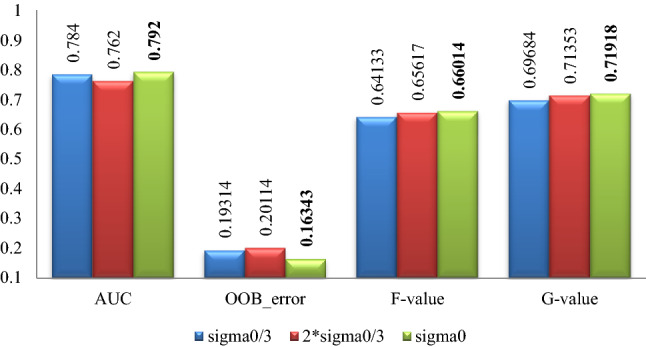
WPBC experimental result graph.

**Figure 9:**
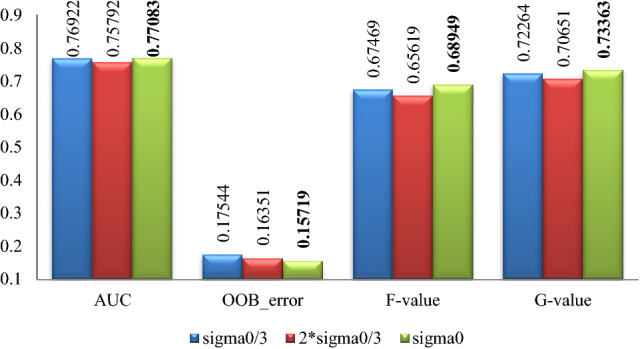
Ionosphere experimental result graph.

**Figure 10:**
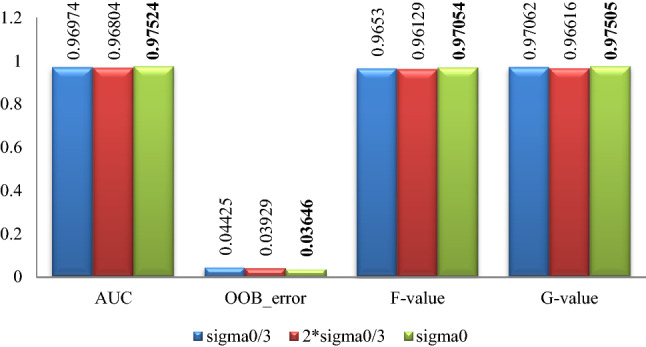
Breast-cancer-wisconsin experimental result graph.

**Figure 11:**
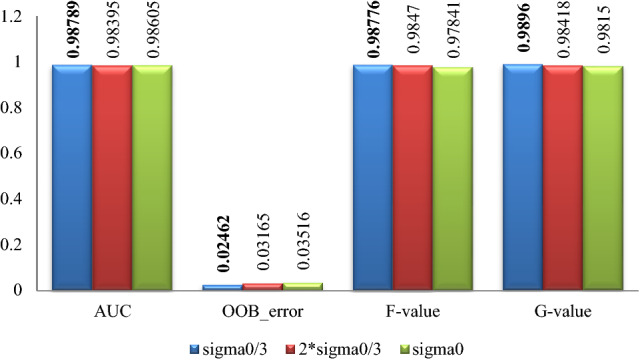
WDBC experimental result graph.

The classification effect by random forest after data expanding by proposed algorithm with different parameters for the 5 datasets is summarized in Figs. 7, 8, 9, 10, 11, where Sigma0 ($\sigma_{0}$) denotes the standard deviation of the original minority normalized data. The bold font represents the experimental results are the best ones.

According to Figs. 7, 8, 9, 10, 11, for the Pima data set, the classification effect of random forest is generally better when the parameter $\sigma$ of the Normal distribution in Eq. (5) takes $\sigma = \sigma_{0}$, although the corresponding *F*-value is higher when $\sigma = {{2\sigma_{0} } \mathord{\left/ {\vphantom {{2\sigma_{0} } 3}} \right. \kern-\nulldelimiterspace} 3}$; For the WPBC, Ionosphere, and Breast-cancer-wisconsin data sets, the classification effect of random forest is the best when the parameter $\sigma$ takes $\sigma = \sigma_{0}$; For WDBC data set, the classification effect of random forest is the best when the parameter $\sigma$ takes $\sigma = {{\sigma_{0} } \mathord{\left/ {\vphantom {{\sigma_{0} } 3}} \right. \kern-\nulldelimiterspace} 3}$ in the improved SMOTE algorithm.

### Part 3 experimental results

The experimental results of part3 are shown in Tables 3, 4. Where bold font indicates the best experimental results.

**Table 3:** Inter-class distance.

	Pima	WPBC	Breast	Ionosphere	WDBC
Original normalization	0.257237	0.553262	1.439431	0.619462	1.741354
$\sigma = \sigma_{0}$	**0.257311**	**0.552654**	**1.439469**	0.620011	1.742575
$\sigma = {{2\sigma_{0} } \mathord{\left/ {\vphantom {{2\sigma_{0} } 3}} \right. \kern-\nulldelimiterspace} 3}$	0.257054	0.552475	1.438660	**0.619066**	1.708176
$\sigma = {{\sigma_{0} } \mathord{\left/ {\vphantom {{\sigma_{0} } 3}} \right. \kern-\nulldelimiterspace} 3}$	0.256641	0.554052	1.440172	0.624840	**1.741331**

**Table 4:** Sample variance table.

	Pima	WPBC	Breast	Ionosphere	WDBC
Original normalization	0.031977	0.053232	0.094468	0.104783	0.059669
$\sigma = \sigma_{0}$	**0.024752**	**0.027806**	**0.074623**	**0.085436**	0.051303
$\sigma = {{2\sigma_{0} } \mathord{\left/ {\vphantom {{2\sigma_{0} } 3}} \right. \kern-\nulldelimiterspace} 3}$	0.024581	0.026953	0.073458	0.084582	**0.054855**
$\sigma = {{\sigma_{0} } \mathord{\left/ {\vphantom {{\sigma_{0} } 3}} \right. \kern-\nulldelimiterspace} 3}$	0.024493	0.026554	0.072783	0.083650	0.050742

According to Table 3, it can be found that for the Pima, WPBC and Breast-cancer-wisconsin datasets, when the standard deviation $\sigma$ of the Normal distribution in Eq. (5) takes $\sigma = \sigma_{0}$, the inter-class distance between categories after expanded is closest to that of the original unexpanded data. For the Ionosphere data set, when the standard deviation $\sigma$ takes $\sigma = {{2\sigma_{0} } \mathord{\left/ {\vphantom {{2\sigma_{0} } 3}} \right. \kern-\nulldelimiterspace} 3}$, the inter-class distance between categories after expanded is closest to that of the original data, and it is quite closer when takes $\sigma = \sigma_{0}$. For the WDBC data set, when the standard deviation $\sigma$ takes $\sigma = {{\sigma_{0} } \mathord{\left/ {\vphantom {{\sigma_{0} } 3}} \right. \kern-\nulldelimiterspace} 3}$, the inter-class distance between categories after expanded is closest to that of the original unexpanded data.

According to Table 4 it can be found that for the Pima, WPBC, Ionosphere, and Breast-cancer-wisconsin datasets, when the standard deviation $\sigma$ of the Normal distribution in Eq. (5) takes $\sigma = \sigma_{0}$, the sample variance of the expanded data is the closest to that of the original unexpanded data. For the WDBC datasets, When the standard deviation $\sigma$ takes $\sigma = {{2\sigma_{0} } \mathord{\left/ {\vphantom {{2\sigma_{0} } 3}} \right. \kern-\nulldelimiterspace} 3}$, the sample variance of the expanded data is the closest to that of the original unexpanded data.

Combining with the experimental results in Figs. 7, 8, 9, 10, 11, it can be seen that for Pima, WPBC, and Breast-cancer-wisconsin dataset, the corresponding classification effect is the best when takes the parameter (Normal distribution standard deviation) of $\sigma$ as $\sigma = \sigma_{0}$ in Eq. (5) to expand the data set. And the inter-class distance and sample variance after expansion are the closest to that of the original data in this condition. It suggests that the better the distribution characteristics of the original minority data are maintained, the more the expended data is similar to the original minority data, then the better the classification effect is. For the Ionosphere and WDBC dataset, the data do not show a consistent pattern. While for Ionosphere, it is not so confusing to get that the corresponding classification effect is the best and statistical characteristics are well maintained under the parameter selection $\sigma = \sigma_{0}$, because the sample variance value is the closest to that of the original one, and the inter-class distance between categories is quite similar to that of expansion under the parameter selection $\sigma = {{2\sigma_{0} } \mathord{\left/ {\vphantom {{2\sigma_{0} } 3}} \right. \kern-\nulldelimiterspace} 3}$. For the WDBC data set, the inter-class distance between categories after expansion is closest to that of the original condition when $\sigma = {{\sigma_{0} } \mathord{\left/ {\vphantom {{\sigma_{0} } 3}} \right. \kern-\nulldelimiterspace} 3}$, while the variance of the extended data is closest to that of the original unexpanded data when $\sigma = {{2\sigma_{0} } \mathord{\left/ {\vphantom {{2\sigma_{0} } 3}} \right. \kern-\nulldelimiterspace} 3}$, it is confusing to make parameter selection. Considering the nature of the classification problem, it is clear that the data set is more separable when the inter-class distance between the categories is greater and the divergence within the classes is smaller. Then it is not hard to choose the parameter $\sigma = {{\sigma_{0} } \mathord{\left/ {\vphantom {{\sigma_{0} } 3}} \right. \kern-\nulldelimiterspace} 3}$ to get expansion data with closest inter-class distance to original condition and a smaller divergence.

The experimental results reveal a clue that the parameters selected when the statistical characteristics of the expanded data are closer to that of the original data are optimal. To verify the conclusion more rigorously, more detailed options for parameter selection should be considered in future.

## Conclusion

Aiming at the problem of classification of imbalanced data sets, a new data expansion algorithm based on the idea of Normal distribution is proposed in this paper. The algorithm expands the minority data by linear interpolation based on the Normal distribution trend between the minority sample points and the minority center, so that the newly generated minority data distributed closer to the center of the minority sample with a higher probability to effectively expand minority samples and avoid marginalization. The experiments show that a better classification effect could be got when the proposed algorithm is used to expand the five imbalance datasets than that of the condition of original SMOTE algorithm. In addition, the inter-class distance and sample variance of augmented data by the proposed algorithm with different parameters ($\sigma = \sigma_{{0}}$,$\sigma = {{2\sigma_{{0}} } \mathord{\left/ {\vphantom {{2\sigma_{{0}} } 3}} \right. \kern-\nulldelimiterspace} 3}$ and $\sigma = {{\sigma_{{0}} } \mathord{\left/ {\vphantom {{\sigma_{{0}} } 3}} \right. \kern-\nulldelimiterspace} 3}$) are calculated, and the comparison of the classification effect of the random forests are analyzed. It is revealed that when the inter-class distance and sample variance of the expanded data are closer to those of the original data, the classification effect of the random forest is the best in the designed experiments.

## Data Availability

The data used to support the results of this study is publicly available and can be obtained from the website http://archive.ics.uci.edu/ml/.

## References

[1] Qinghua H, Gui Changqing Xu, Jie LG (2019). A generalized method to predict the compressive strength of high-performance concrete by improved random forest algorithm. Constr. Build. Mater..

[2] Verbiest N, Ramentol E, Cornelis C, Herrera F (2014). Preprocessing noisy imbalanced datasets using SMOTE enhanced with fuzzy rough prototype selection. Appl. Soft Comput..

[3] Huang L, Fu Q, Li G, Luo B, Chen D, Yu H (2019). Improvement of maximum variance weight partitioning particle filter in urban computing and intelligence. IEEE Access.

[4] Huang L, Fu Q, He M, Jiang D, Hao Z (2021). Detection algorithm of safety helmet wearing based on deep learning. Concurr. Comput. Pract. Exp..

[5] Yu M, Li G, Jiang D, Jiang G, Tao B, Chen D (2019). Hand medical monitoring system based on machine learning and optimal EMG feature set. Pers. Ubiquit. Comput..

[6] Cao Q, Zhang W, Zhu Y (2021). Deep learning-based classification of the polar emotions of “Moe”-Style cartoon pictures. Tsinghua Sci. Technol..

[7] Palmer J, Sheng VS, Atkison T (2020). Classification on grade, price, and region with multi-label and multi-target methods in wineinformatics. Big Data Min. Anal..

[8] Guezzaz A, Asimi Y, Azrour M (2021). Mathematical validation of proposed machine learning classifier for heterogeneous traffic and anomaly detection. Big Data Min. Anal..

[9] Kam J, Dick S (2006). Comparing nearest-neighbour search strategies in the SMOTE algorithm. Can. J. Electr. Comput. Eng..

[10] Demidova L, Klyueva I (2017). Improving the classification quality of the SVM classifier for the imbalanced datasets on the base of ideas the SMOTE algorithm. Int. Jt. Conf. Mater. Sci. Mech. Eng. (CMSME).

[11] Galar M, Fernández A, Barrenechea E, Herrera F (2013). EUSBoost: Enhancing ensembles for highly imbalanced data-sets by evolutionary undersampling. Pattern Recognit..

[12] Datta S, Das S (2015). Near-Bayesian support vector machines for imbalanced data classification with equal or unequal misclassification costs. Neural Netw..

[13] Yun Q, Yanchun L, Li Mu, Guoxiang F, Xiaohu S (2014). A resampling ensemble algorithm for classification of imbalance problems. Neurocomputing.

[14] Yijing C, Bo P, Guolin S, Guozhu W, Xingshu C (2021). DGA-based botnet detection toward imbalanced multiclass learning. Tsinghua Sci. Technol..

[15] Hou C, Jiawei Wu, Cao B, Fan J (2021). A deep-learning prediction model for imbalanced time series data forecasting. Big Data Min. Anal..

[16] Nitesh VC, Kevin WB, Lawrence OH (2002). SMOTE: Synthetic minority over-sampling technique. J. Artif. Intell. Res..

[17] Blagus R, Lusa L (2013). SMOTE for high-dimensional class-imbalanced data. BMC Bioinf..

[18] Mi Y (2013). Imbalanced classification based on active learning SMOTE. Res. J. Appl. Sci Eng. Technol..

[19] Seo JH, Kim YH (2018). Machine-learning approach to optimize SMOTE ratio in class imbalance dataset for intrusion detection. Comput. Intell. Neurosci..

[20] Guo, S., Liu, Y. & Chen, R. *et al*. Improved SMOTE algorithm to deal with imbalanced activity classes in smart homes. *Neural Process. Lett.* 1–24.

[21] Yang, L., Li, P. & Xue, R. *et al*. Intelligent classification model for railway signal equipment fault based on SMOTE and ensemble learning. *International Joint Conference on Materials Science and Mechanical Engineering (CMSME)*383 (2018): 1–9.

[22] Douzas G, Bacao F (2019). Geometric SMOTE a geometrically enhanced drop-in replacement for SMOTE. Inf. Sci..

[23] Li Ma, Suohai F (2017). CURE-SMOTE algorithm and hybrid algorithm for feature selection and parameter optimization based on random forests. BMC Bioinf..

[24] Prusty MR, Jayanthi T, Velusamy K (2017). Weighted-SMOTE: A modification to SMOTE for event classification in sodium cooled fast reactors. Prog. Nucl. Energy.

[25] Xwl, A., Apj A. & Tl, A. *et al*. LR-SMOTE—An improved unbalanced data set oversampling based on K-means and SVM. Knowledge-Based Systems 196 (2020).

[26] Fernandez A, Garcia S, Chawla NV (2018). SMOTE for learning from imbalanced data: Progress and challenges, marking the 15-year anniversary. J. Artif. Intell. Res..

[27] Majzoub HA, Elgedawy I, Akaydn Y (2020). HCAB-SMOTE: A hybrid clustered affinitive borderline SMOTE approach for imbalanced data binary classification. Arab. J. Sci. Eng..

[28] Chen B, Xia S, Chen Z (2020). RSMOTE: A self-adaptive robust SMOTE for imbalanced problems with label noise. Inf. Sci..

[29] Pescim RR, Clarice GB, Demétrio CGM (2010). The beta generalized half-normal distribution. Comput. Stat. Data Anal..

[30] Flacke SJ, Fischer SE, Lorenz CH (2001). Measurement of the Gadopentetate Dimeglumine partition coefficient in human myocardium in vivo: Normal distribution and elevation in acute and chronic infarction1. Radiology.

[31] Breiman L (2001). Random forest. Mach. Learn..

[32] Hong, J.-S. Microstrip filters for RF/microwave applications. *IEEE Microwave Mag.* **3**(3), 62–65 (2002).

[33] Svetnik, V. Random forest: A classification and regression tool for compound classification and QSAR modeling. *J. Chem. Inf. Comput. Sci*., **43** (2003). 10.1021/ci034160g 14632445

[34] Strobl, C., Boulesteix, A. L. & Zeileis, A. *et al.* Bias in random forest variable importance measures: Illustrations, sources and a solution. *BMC Bioinf.* **8**, (2007). 10.1186/1471-2105-8-25 PMC1796903 17254353

[35] Tan Xiaopeng Su, Shaojing HZ, Xiaojun G, Zhen Z, Xiaoyong S, Longqing Li (2019). Wireless sensor networks intrusion detection based on SMOTE and the random forest algorithm. Sensors (Basel, Switzerland).

